# The role of cochlear implant positioning on MR imaging quality: a preclinical in vivo study with a novel implant magnet system

**DOI:** 10.1007/s00405-021-07005-y

**Published:** 2021-08-09

**Authors:** Pietro Canzi, Marianna Magnetto, Anna Simoncelli, Marco Manfrin, Federico Aprile, Elvis Lafe, Elena Carlotto, Irene Avato, Andrea Scribante, Lorenzo Preda, Marco Benazzo

**Affiliations:** 1 Department of Clinical, Surgical, Diagnostic and Pediatric Sciences, University of Pavia, Pavia, Italy; 2 Department of Otorhinolaryngology, Fondazione IRCCS Policlinico San Matteo, University of Pavia, Viale Camillo Golgi, 19, 27100 Pavia, Italy; 3 Department of Diagnostic Radiology and Interventional Radiology and Neuroradiology, Fondazione IRCCS Policlinico San Matteo, University of Pavia, Pavia, Italy; 4 PhD in Experimental Medicine, University of Pavia, Pavia, Italy

**Keywords:** Cochlear implant, Implant positioning, MRI artifacts, Rotatable internal magnets, Diametrically bipolar magnets

## Abstract

**Purposes:**

To investigate the effects for Ultra 3D cochlear implant (CI) positioning on MR imaging quality, looking at a comprehensive description of intracranial structures in cases of unilateral and bilateral CI placement.

**Methods:**

Four CI angular positions (90°, 120°, 135° and 160°) at 9 cm distance from the outer-ear canal were explored. The 1.5 T MRI assessment included our institutional protocol for the investigation of brain pathologies without gadolinium application. Three investigators (two experienced neuroradiologists and one experienced otoneurosurgeon) independently evaluated the MR findings. A 4-point scale was adopted to describe 14 intracranial structures and to determine which CI positioning allowed the best image quality score and how bilateral CI placement modified MRI scan visibility.

**Results:**

A high positive correlation was found between the three blinded observers. Structures situated contralateral from the CI showed high-quality values in all four placements. Structures situated ipsilaterally provided results suitable for diagnostic purposes for at least one position. At 90°, artifacts mainly involved brain structures located cranially and anteriorly (e.g., temporal lobe); on the contrary, at 160°, artifacts mostly influenced the posterior fossa structures (e.g., occipital lobe). For the bilateral CI condition, MR imaging examination revealed additional artifacts involving all structures located close to either CI, where there was a signal void/distortion area.

**Conclusions:**

Suitable unilateral CI positioning can allow the visualization of intracranial structures with sufficient visibility for diagnostic purposes. Bilateral CI positioning significantly deteriorates the anatomical visibility. CI positioning might play a crucial role for patients who need post-operative MRI surveillance.

**Supplementary Information:**

The online version contains supplementary material available at 10.1007/s00405-021-07005-y.

## Introduction

Over time, the MRI compatibility issue has become one of the most relevant challenges faced by years of cochlear implant (CI) technological research. Pain, magnet migration, reversal of magnet polarity are possible adverse events related to CI-MRI interaction [1]. To overcome these limitations, new generations of CIs with self-aligning magnets have been developed. In 2014, Med-El (Med-El, Innsbruck, Austria), first designed a CI model with a freely rotating magnet in one axis. More recently, Advanced Bionics AG (AB—Stäfa, Switzerland), released a new CI model (HiRes™ Ultra 3D) featuring four independent magnet bars free to rotate on two axes. Manufacturers’ guidelines indicate MRI compatibility up to 3 T without the need for magnet removal or head bandaging [2]. Once those MRI safety issues have been satisfied, new considerations need to be addressed. Patients previously precluded from cochlear implantation, because of the need for ongoing MRI surveillance, may now be candidates for CI surgery. For these patients it is necessary to evaluate the possibility of visualizing the particular intracranial structures of interest. The depiction of anatomical structures mainly depends on the location and extent of signal void and distortion area produced by the interaction between the internal CI magnet system and the MR field. Viable strategies to handle artifact effects include internal magnet removal [3], head orientation [4, 5], MRI algorithms manipulation [6–8] and CI positioning [9]. Until now, only two studies (performed by the same team of researchers) have investigated the influence of CI positioning with non-rotatable internal magnets on MRI artifacts. Both studies, focused on posterior fossa visibility [9, 10]. The aim of the present study was to investigate the effects of Ultra 3D CI position on MR imaging quality, looking at a comprehensive description of intracranial structures in the case of either unilateral or bilateral implantation.

## Materials and methods

**Fig. 1:**
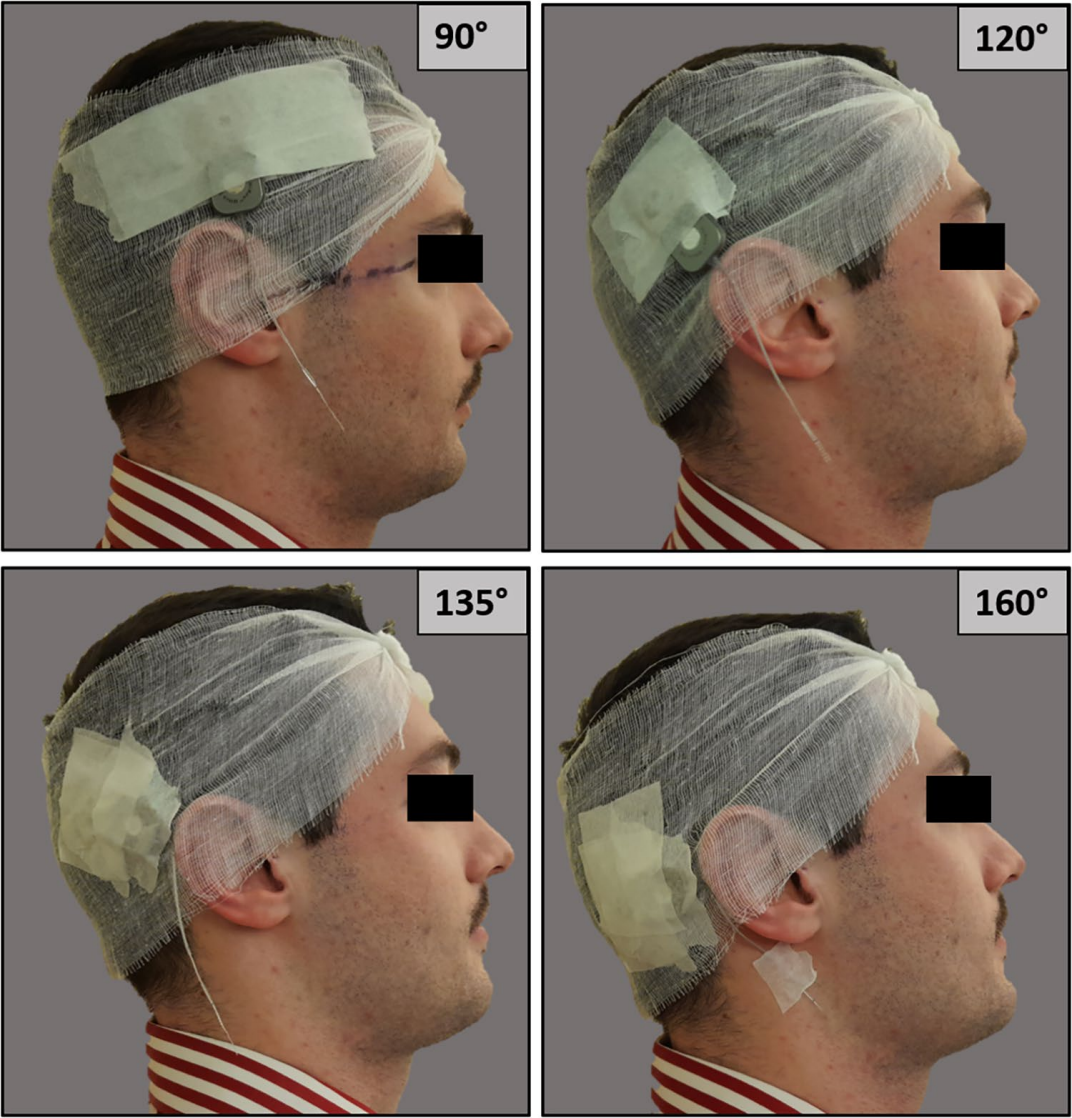
CI positioning on healthy volunteer (90°, 120°, 135°, 160°)

The study was conducted on two healthy adult male volunteers who offered written informed consent after approval from the institutional review board and medical ethics committees. Two AB HiRes™ Ultra 3D CIs with Slim J electrode arrays were supplied for research purposes. A thin single-layer medical gauze was tied to the volunteer’s head. The implant package, with its magnet system in place, was then placed onto the head of each volunteer and held in place by a medical patch. No bandage, or polyethene block were used to restrain the implant package. The CI package’s location was determined with respect to the nasion-outer-ear canal line, the package being oriented in this direction, with the center of the magnet located 9 cm behind the outer-ear canal. Four angles (90°, 120°, 135° and 160°) of orientation were explored (Fig. 1) for both unilateral and bilateral placement.

### Imaging study protocol

All MRI examinations were carried out using the Ingenia™ (Philips Medical Systems, Best, Netherlands) 1.5 T MRI scanner. The MRI assessment included our institutional protocol (without gadolinium) for the investigation of brain pathologies. The acquisition protocol involved planar T1 and T2 weighted (w) turbo spin echo sequences. In particular, the following parameters were selected:

- Axial T1w: repetition time (TR) 550 ms, echo time (TE) 10 ms, slice thickness 2.5 mm, field of view (FoV) 120 × 179 mm^2^, acquisition time 3:02 min; coronal T1w: TR 550 ms, TE 10 ms, slice thickness 2.5 mm, FoV 120 × 179 mm^2^, acquisition time 2:23 min.
–Axial T2w: TR 3000 ms, TE 120 ms, slice thickness 3 mm, FoV 150 × 169 mm^2^, acquisition time 4:36 min; coronal T2w: TR 3036 ms, TE 120 ms, slice thickness 2.5 mm, FoV 120 × 179 mm^2^, acquisition time 3:51 min.

The acquisition protocol was completed with volumetric T1w turbo fast echo sequences (TR 14 ms, TE 6.5 ms, slice thickness 1.1 mm, FoV 256 × 240 mm^2^, acquisition time 5:17 min).

### Diagnostic usefulness analysis

Three investigators (two experienced neuroradiologists and one experienced otoneurosurgeon) independently evaluated the MR findings. A 4-point scale (0 = completely unusable, 1 = visible but not suitable for diagnostic purposes due to artifact contamination, 2 = contaminated by artifact but adequate for diagnostic purposes, 3 = high-quality image of the anatomic structure) was adopted to describe 14 intracranial structures: frontal lobe, parietal lobe, temporal lobe, occipital lobe, hypophysis, internal auditory canal, cochlea, semi-circular canals, vestibulum, brainstem, anterior lobe of the cerebellum, cerebellar vermis, middle cerebellar pedunculus and the cerebellopontine angle. Ipsilateral and contralateral structures, with respect to the CI side, were examined. When unpaired median structures were described (e.g., hypophysis, brainstem, cerebellar vermis), both ipsi- and contralateral sides of each structure were evaluated. Finally, MRI findings were analyzed to investigate specific questions:

- Which CI position allows the best image quality score referring to a specific anatomical structure?
- Which anatomical structure modifies its MRI visibility under bilateral cochlear placement according to each CI position?
- Which CI position allows the best global visualization of the intracranial structures?

### Statistical analysis

**Fig. 2:**
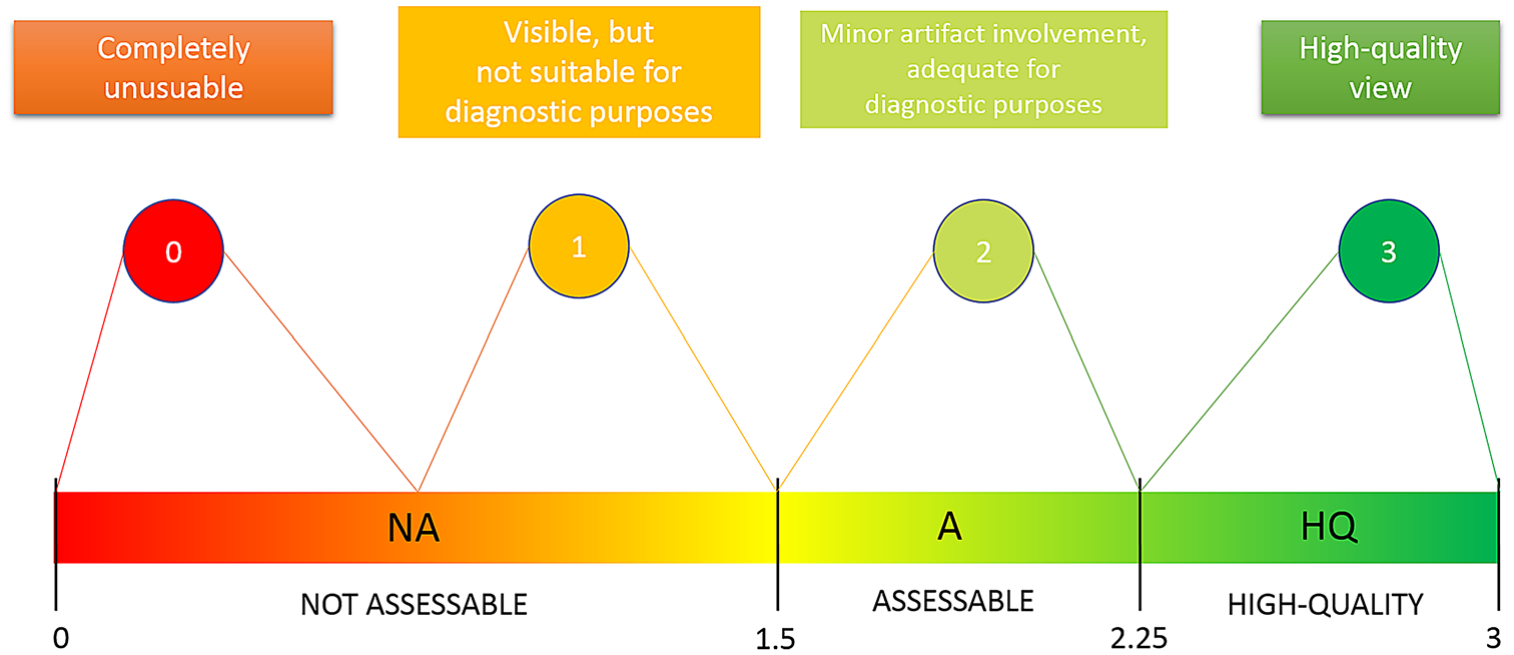
Graphical view of the diagnostic usefulness analysis

Statistical analyses were performed using the R software (R version 3.1.3, R Development Core Team, R Foundation for Statistical Computing, Wien, Austria). Descriptive statistics were calculated for all groups (the mean, standard deviation, median, minimum and maximum values). A Kruskal Wallis test was applied to determine whether significant differences existed between the groups. Dunn’s multiple comparisons test was used as a post-hoc evaluation. The limit for statistical significance for all statistical tests was predetermined at *p* < 0.05. Diagnostic validity of each structure was described using the following ranges (Fig. 2):

o 0 ≤ X < 1.5: not assessable (NA)
p 1.5 ≤ X < 2.25: involved by artifact but assessable for diagnostic purposes (A)
q X ≥ 2.25: high-quality (HQ)

Inter-rater reliability was assessed using a Spearman r correlation.

## Results

Inter-rater reliability agreement ranged from 0.73 to 0.86 across all image evaluations and was consistent with “high positive correlation” among the three investigators. Volunteers reported no subjective discomfort (e.g., pressure on the side of the magnet, pain, magnet displacement) during the MRI examinations.

**Fig. 3:**
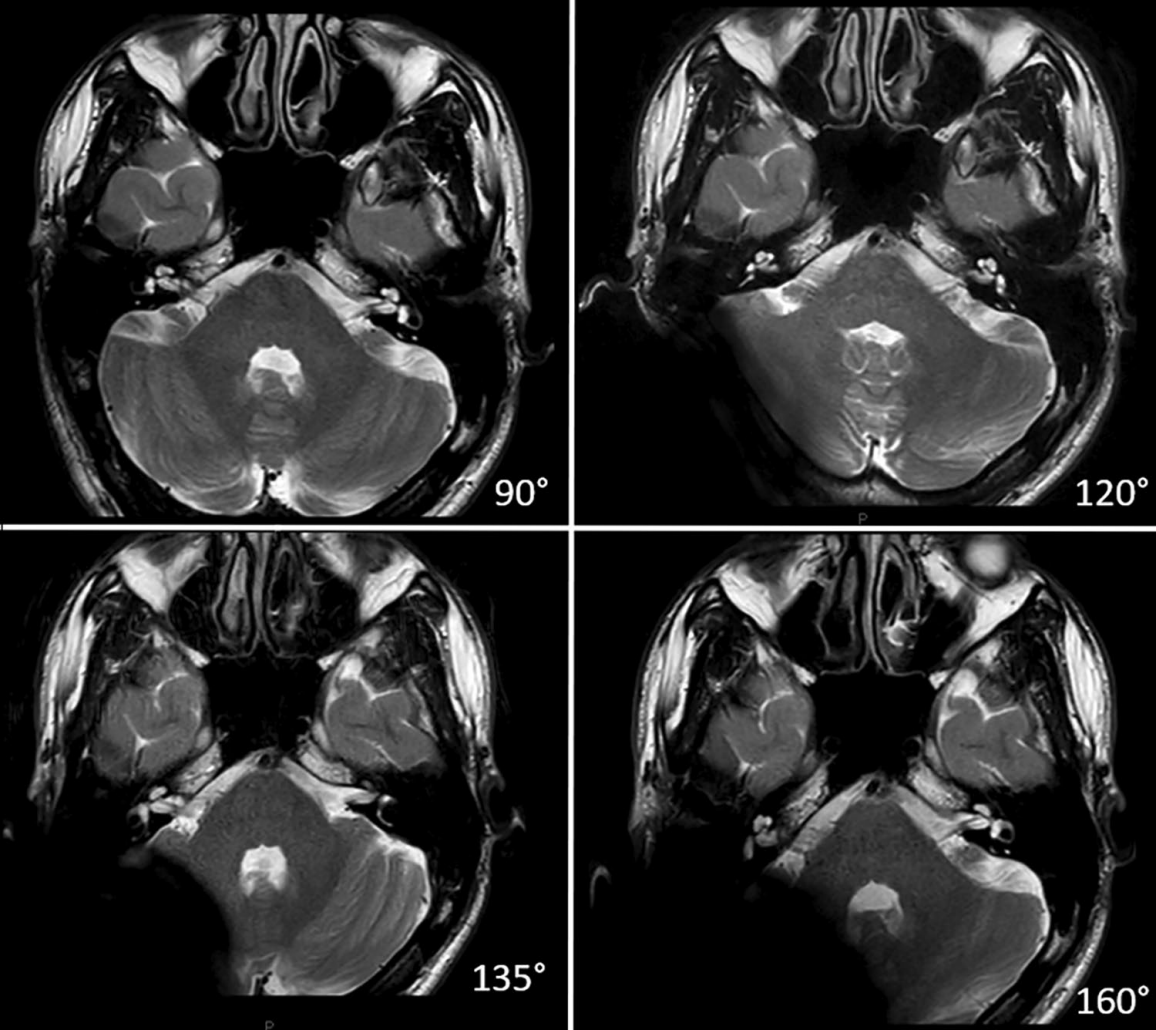
Ipsilateral inner ear and internal auditory canal MRI visibility according to each CI position on 2D axial T2w sequences

**Fig. 4:**
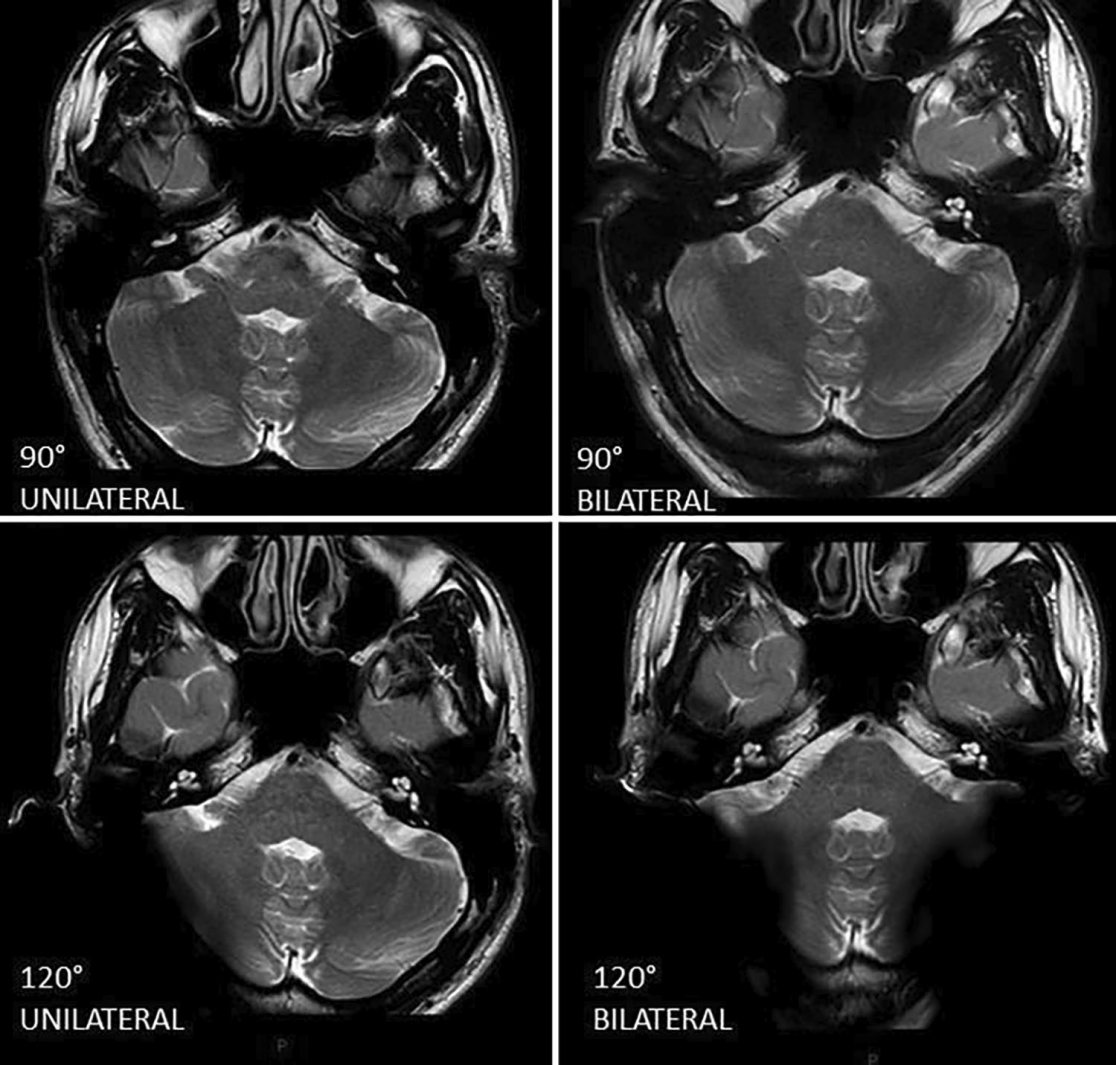
Cerebellar vermis visibility according to 90° and 120° unilateral and bilateral CI positions. When 90° CI positioning was evaluated, the cerebellar vermis appeared highly visible for both unilateral and bilateral CI placement. When 120° CI positioning was considered, the cerebellar vermis visibility was highly assessable in unilateral cochlear placement; however, it was only assessable in case of bilateral CI placement

**Fig. 5:**
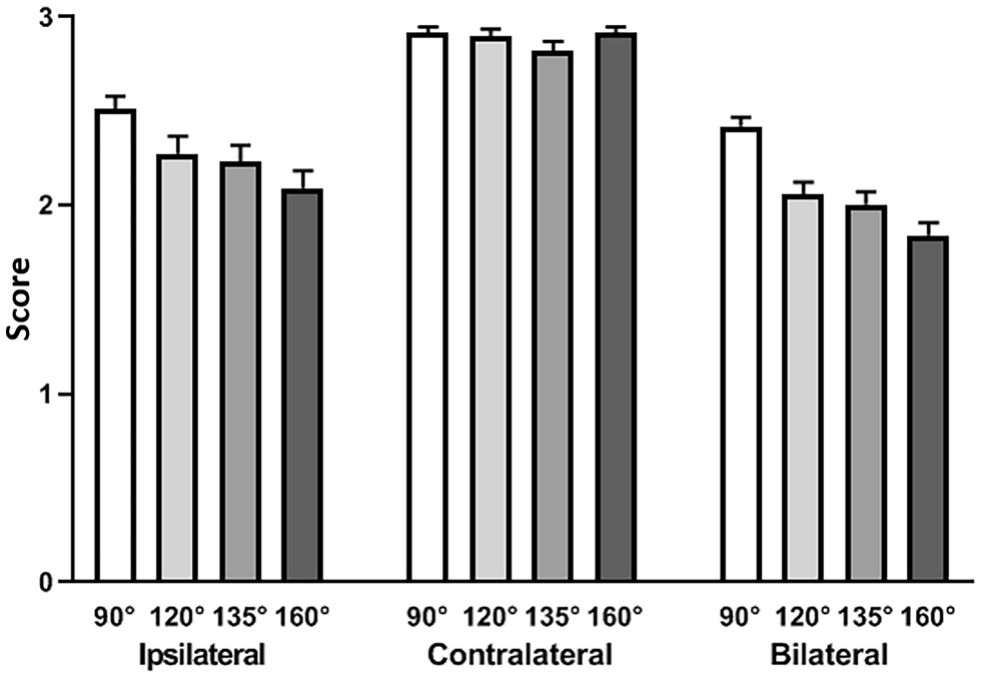
Global anatomical visibility assessment. Global mean scores and confidence intervals according to each CI position and condition: unilateral (ipsilateral and contralateral structures visibility), bilateral cochlear placement

- ***Which CI positioning allows the best image quality score referring to a specific anatomical structure?***
  When ipsilateral structures were examined under unilateral CI positioning, all 14 structures came out as assessable for diagnostic purposes in at least one CI position (Table 1). Moreover, view quality appeared highly assessable for 11 structures in at least one CI position. The occipital lobe achieved the lowest mean quality score (0) while the hypophysis scored the highest (3). When each anatomical structure was considered, CI positioning significantly modified (*p* < 0.05) the image quality scores as follows:
- frontal lobe: 90° vs. 160°
- occipital lobe: 90° vs. 120°/90° vs. 135°/90° vs 160°
- semi-circular canals: 90° vs. 160°
- vestibulum: 90° vs. 160° (Fig. 3)
- anterior lobe cerebellum: 90° vs. 120°/90° vs. 135°/90° vs. 160°
- cerebellar vermis: 90° vs. 120°/90° vs. 135°/90° vs. 160°/120° vs. 160°/135° vs. 160°
- middle cerebellar peduncle: 90° vs. 160°/120° vs. 160°/135° vs. 160°
  Structures sided contralateral from the CI showed HQ values in all four placements (Table 2). Differences among alternative placements resulted statistically significant (*p* < 0.05) for the cerebellar vermis only (90° vs. 160°).
- Which anatomical structure modifies its MRI visibility under bilateral cochlear placement according to each CI position?
  When each anatomical structure was analyzed under bilateral cochlear placement, CI positioning significantly modified (*p* < 0.05) the image quality rating as observed under unilateral CI. In addition to these findings, a further artifact was observed involving all anatomical structures close to the signal void/distortion area produced by each CI in case of bilateral cochlear placement. Consequently, when ipsilateral structures were compared under for both unilateral and bilateral cases, visibility was significantly modified (*p* < 0.05) for the following CI positions (Table 3; Fig. 4):
- 90°: parietal lobe and brainstem.
- 120°: semi-circular canals, vestibulum, brainstem, anterior lobe of the cerebellum, cerebellar vermis, middle cerebellar peduncle.
- 135°: parietal lobe, cochlea, brainstem, cochlea, cerebellar vermis, middle cerebellar peduncle, cerebellopontine angle.
- 160°: frontal lobe, internal auditory canal, brainstem, anterior lobe of the cerebellum, cerebellar vermis, middle cerebellar peduncle, cerebellopontine angle.
  No statistically significant differences were found when the left and right structures were compared (*p* > 0.05) in case of bilateral cochlear placement.
  An MRI diagnostic usefulness atlas considering cochlear implant positioning is supplied as supplementary material.
- Which CI positioning allows the best global visualization of the intracranial structures?
  When all 14 intracranial structures ipsilateral to cochlear implantation were globally evaluated, 90° and 120° CI positioning allowed better image quality scores than 135° (*p* > 0.05) and 160° CI placement (*p* < 0.05; Table 4). No statistical differences were found when contralateral structures were analyzed according to each CI position (*p* > 0.05). In case of bilateral CI, surgical positioning significantly modified the image quality ratings as follows (*p* < 0.05): 90° vs. 120°; 90° vs. 135°; 90° vs. 160°; 120° vs. 160°; 135° vs. 160°. Overall, the 90° CI placement produced the best global image quality rating (*p* < 0.05; Fig. 5).

**Table 1:** Anatomical visibility assessment: unilateral CI–ipsilateral structures

	Unilateral CI			
Ipsilateral structures	90° (Mean–SD)	120° (Mean–SD)	135° (Mean–SD)	160° (Mean–SD)
Frontal lobe	A (2.10–0.31)	A (2.23–0.50)	HQ (2.33–0.48)	HQ (2.53–0.51)
Parietal lobe	A (1.70–0.60)	A (1.67–0.71)	A (1.80–0.41)	A (1.73–0.58)
Temporal lobe	A (1.77–0.63)	A (1.80–0.61)	A (1.90–0.31)	A (2.00–0)
Occipital lobe	A (1.83–0.59)	NA (0.33–0.48)	NA (0.20–0.41)	NA (0–0)
Hypophysis	HQ (2.90–0.31)	HQ (3–0)	HQ (2.97–0.18)	HQ (3– 0)
Internal auditory canal	HQ (2.83–0.53)	HQ (2.63–0.77)	HQ (2.67–0.66)	HQ (2.77–0.63)
Cochlea	HQ (2.83–0.53)	HQ (2.67–0.71)	HQ (2.70–0.65)	HQ (2.73–0.64)
Semicircular canals	HQ (2.57–0.68)	HQ (2.47–0.82)	A (2.17–0.80)	A (1.90–1.0)
Vestibulum	HQ (2.73–0.64)	HQ (2.63–0.77)	HQ (2.47–0.82)	A (2.17–1.02)
Brainstem	HQ (2.90–0.31)	HQ (2.80–0.61)	HQ (2.80–0.48)	HQ (2.83–0.38)
Anterior lobe of the cerebellum	HQ (2.63–0.77)	A (2.00–0.83)	A (1.87–0.73)	A (1.67–0.66)
Cerebellar vermis	HQ (2.80–0.61)	HQ (2.27–0.79)	A (2.23–0.73)	NA (1.47–0.73)
Middle cerebellar peduncle	HQ (2.80–0.61)	HQ (2.70–0.65)	HQ (2.47–0.78)	A (1.77–0.68)
Cerebellopontine angle	HQ (2.77–0.57)	HQ (2.67–0.71)	HQ (2.67–0.71)	HQ (2.70–0.70)

*HQ* high-quality image; A: image involved by artifact, but assessable for diagnostic purposes; *NA* not assessable image; *SD* standard deviation

Mean and standard deviation referred to the image quality scores assessed by all investigators according to all MRI sequences for ipsilateral structures, with respect to the CI side

**Table 2:** Anatomical visibility assessment: unilateral CI–contralateral structures

Contralateral structures	Unilateral CI			
	90° (Mean–SD)	120° (Mean–SD)	135° (Mean–SD)	160° (Mean–SD)
Frontal lobe	HQ (2.80–0.40)	HQ (2.90–0.30)	HQ (2.90–0.30)	HQ (3–0)
Parietal lobe	HQ (2.80–0.40)	HQ (2.90–0.30)	HQ (2.90–0.40)	HQ (2.90–0.30)
Temporal lobe	HQ (2.90–0.30)	HQ (2.90–0.30)	HQ (3–0.2)	HQ (3–0)
Occipital lobe	HQ (2.90–0.30)	HQ (2.70–0.70)	HQ (2.60–0.70)	HQ (2.60–0.60)
Hypophysis	HQ (2.90–0.30)	HQ (3–0)	HQ (3–0.20)	HQ (3–0)
Internal auditory canal	HQ (3–0.20)	HQ (3–0)	HQ (2.80–0.60)	HQ (3–0)
Cochlea	HQ (3–0)	HQ (3–0)	HQ (2.80–0.60)	HQ (3–0)
Semicircular canals	HQ (3–0)	HQ (3–0)	HQ (2.80–0.60)	HQ (3–0)
Vestibulum	HQ (3–0.20)	HQ (3–0)	HQ (2.80–0.60)	HQ (3–0)
Brainstem	HQ (2.90–0.30)	HQ (2.80–0.50)	HQ (2.90–0.40)	HQ (3–0)
Anterior lobe of the cerebellum	HQ (3–0.20)	HQ (2.90–0.30)	HQ (2.80–0.60)	HQ (2.90–0.30)
Cerebellar vermis	HQ (2.80–0.60)	HQ (2.70–0.70)	HQ (2.60–0.80)	HQ (2.40–0.90)
Middle cerebellar peduncle	HQ (2.90–0.30)	HQ (2.80–0.60)	HQ (2.80–0.60)	HQ (3–0)
Cerebellopontine angle	HQ (3–0)	HQ (3–0)	HQ (2.90–0.40)	HQ (3–0)

*HQ* high-quality image; A: image involved by artifact, but assessable for diagnostic purposes; *NA* not assessable image; *SD* standard deviation

Mean and standard deviation referred to the image quality scores assessed by all investigators according to all MRI sequences for contralateral structures, with respect to the CI side

**Table 3:** Anatomical visibility comparison between unilateral and bilateral CI

	90° (Mean–SD)		120° (Mean–SD)		135° (Mean–SD)		160° (Mean–SD)	
	Unilateral	Bilateral	Unilateral	Bilateral	Unilateral	Bilateral	Unilateral	Bilateral
Frontal lobe	A (2.10–0.31)	A (2.10–0.30)	A (2.23–0.50)	A (2.21–0.50)	HQ (2.33–0.48)	A (2.20–0.61)	HQ (2.53–0.51)	HQ (2.33–0.48)
Parietal lobe	A (1.70–0.60)	NA (1.40–0.62)	A (1.67–0.71)	A (1.53–0.65)	A (1.80–0.41)	A (1.67–0.66)	A (1.73–0.58)	A (1.80–0.40)
Temporal lobe	A (1.77–0.63)	A (1.78–0.45)	A (1.80–0.61)	A (2–0)	A (1.90–0.31)	A (1.78–0.61)	A (2–0)	A (1.93–0.25)
Occipital lobe	A (1.83–0.59)	A (1.83–0.38)	NA (0.33–0.48)	NA (0.28–0.45)	NA (0.20–0.41)	NA (0.23–0.43)	NA (0–0)	NA (0–0)
Hypophysis	HQ (2.90–0.31)	HQ (2.87–0.34)	HQ (3–0)	HQ (3–0)	HQ (2.97–0.18)	HQ (2.80–0.61)	HQ (3–0)	HQ (2.97–0.18)
Internal auditory canal	HQ (2.83–0.53)	HQ (2.72–0.64)	HQ (2.63–0.77)	HQ (2.63–0.76)	HQ (2.67–0.66)	HQ (2.50–0.85)	HQ (2.77–0.63)	HQ (2.47–0.68)
Cochlea	HQ (2.83–0.53)	HQ (2.68–0.68)	HQ (2.67–0.71)	HQ (2.60–0.83)	HQ (2.70–0.65)	HQ (2.42–0.98)	HQ (2.73–0.64)	HQ (2.53–0.65)
Semicircular canals	HQ (2.57–0.68)	HQ (2.50–0.77)	HQ (2.47–0.82)	A (1.92–1.03)	A (2.17–0.80)	A (2.08–1.09)	A (1.90–1)	A (1.72–1.04)
Vestibulum	HQ (2.73–0.64)	HQ (2.57–0.77)	HQ (2.63–0.77)	HQ (2.30–1.17)	HQ (2.47–0.82)	HQ (2.32–1.10)	A (2.17–1.02)	A (1.98–0.95)
Brainstem	HQ (2.90–0.31)	HQ (2.75–0.51)	HQ (2.80–0.61)	HQ (2.33–0.60)	HQ (2.80–0.48)	HQ (2.33–0.75)	HQ (2.83–0.38)	HQ (2.37–0.66)
Anterior lobe of the cerebellum	HQ (2.63–0.77)	HQ (2.57–0.77)	A (2–0.83)	A (1.50–0.68)	A (1.87–0.73)	A (1.62–1.03)	A (1.67–0.66)	NA (1.13–0.62)
Cerebellar vermis	HQ (2.80–0.61)	HQ (2.70–0.53)	HQ (2.27–0.79)	A (1.80–0.55)	A (2.23–0.73)	A (1.67–0.80)	NA (1.47–0.73)	NA (0.90–0.60)
Middle cerebellar peduncle	HQ (2.80–0.61)	HQ (2.68–0.60)	HQ (2.70–0.65)	A (2.02–0.75)	HQ (2.47–0.78)	A (1.93–1.02)	A (1.77–0.68)	NA (1.20–0.78)
Cerebellopontine angle	HQ (2.77–0.57)	HQ (2.72–0.59)	HQ (2.67–0.71)	HQ (2.58–0.77)	HQ (2.67–0.71)	HQ (2.50–1.02)	HQ (2.70–0.70)	HQ (2.43–0.72)

*HQ* high-quality image; A: image involved by artifact, but assessable for diagnostic purposes; *NA* not assessable image; *SD* standard deviation

Mean and standard deviation referred to the image quality scores assessed by all investigators according to all MRI sequences for ipsilateral structures under unilateral and bilateral cochlear placement

**Table 4:** Global anatomical visibility assessment according to each CI positioning

	Ipsilateral (Mean–SD)	Contralateral (Mean–SD)	Bilateral (Mean–SD)
90°	HQ (2.51–0.71)	HQ (2.92–0.30)	HQ (2.42–0.73)
120°	HQ (2.28–0.93)	HQ (2.90–0.38)	A (2.06–0.95)
135°	A (2.23–0.90)	HQ (2.82–0.54)	A (2.00–1.04)
160°	A (2.09–0.98)	HQ (2.91–0.34)	A (1.84–0.99)

*HQ* high-quality image; A: image involved by artifact, but assessable for diagnostic purposes; *NA* not assessable image; *SD* standard deviation

Mean and standard deviation referred to the image quality scores assessed by all investigators according to all intracranial structures and all MRI sequences under unilateral and bilateral cochlear placement

## Discussion

The increasing number of patients undergoing CI surgery, related to an evident expansion of selection criteria [11–14] and an impressive evolution of health care technology [15, 16], has raised many concerns about the issue of CI-MRI compatibility [17, 18]. How best to manage the MRI artifact represents one of the emerging topics related to the new generation of CIs. In the current study, the role of CI position on MR image quality was investigated to describe, for the first time, 14 intracranial structures following unilateral and bilateral CI placement. To more accurately simulate the MR-induced artifacts related to both the metallic CI components and the internal magnet system, we employed two Ultra 3D CIs. A paucity of work deals with MRI artifacts, focused on a few structures’ visibility and involving CI dummies with non-rotatable magnets [9, 10]. In our study, the diagnostic usefulness analysis was provided by three independent observers: with the high positive correlation between them supporting the reliability of the research. Previous studies found it difficult to rate visibility of anatomical structures for 3 T MRI scanning and for a CI distance lower than 9 cm from the outer-ear canal [7, 9, 10]. Moreover, such closer CI positions may lead to uncomfortable use of the “behind-the-ear” sound processor. Accordingly, four rotational CI orientations, each at 9 cm from the outer-ear canal, were investigated in relation to a specific anatomical structure. Each structure analyzed was of diagnostic interest and investigated using a 1.5 T MRI. The results demonstrated that each ipsilateral structure was sufficiently visible for diagnostic purposes in at least one CI position. At 90° orientation, artifacts mainly involved brain structures located cranially and anteriorly (e.g., frontal lobe, temporal lobe); on the contrary, for the 160° orientation, artifacts mostly influenced the MRI view of posterior fossa structures (e.g., occipital lobe, cerebellar vermis). More specifically, when the ipsilateral internal auditory canal and the cochlea were considered, they appeared highly visible for diagnostic purposes in all CI orientations. However, the ipsilateral vestibulum and semi-circular canals showed HQ image scores at 90° and 120° but received mainly assessable image quality ratings at 135° and 160° through being located more posteriorly. These findings were consistent with Todt et al. and Schröeder et al. [9, 10], even if the authors did not make a comparison between each inner ear structure. Interestingly, Todt et al. [9] observed one magnet dislocation and described candidates’ painful pressure during 3 T MRI; Schröeder et al. [10] reported volunteers’ pressure sensations on the side of the magnet during 1.5 T MRI. In contrast, we did not experience any degree of subjective discomfort, in agreement with previous studies involving adaptive magnets [19–22]. The role of CI position on MRI artifact was also investigated for bilateral CI placement. In this condition, MR imaging revealed additional artifacts involving all structures located close to the signal void/distortion area produced by each CI. Posterior fossa structures worsened in their visibility at 160° bilateral CI positioning in comparison with unilateral CI. On the contrary, anterior, and cranial brain structures were more involved by additional artifacts at a 90° bilateral CI position. Overall, the 90-degree orientation provided the best visibility rating concerning the brain structures analyzed, which mainly belonged to the posterior cranial fossa. However, a 90-degree CI position could face daily life problems when wearing caps or headbands, suggesting that in these cases a personalized solution is found. Previous authors have looked at different strategies to handle the impact of MRI artifacts on image quality. Wackym and colleagues [4] found that head rotation angle along the z-axis influences the image degradation produced by the internal non-rotatable magnet. Ay et al. [5] showed that an anteflexion of the head inside the MRI scanner improves the visualization in the coronal plane of the inner auditory canal. Wagner et al. [3] studied the positive effects of magnet removal on image quality; however, a risk-benefits analysis should be carefully pondered concerning the drawbacks of revision surgery. Sharon et al. [6] published the results of acquisition imaging techniques aimed at formulating an MRI protocol to improve image visibility. Finally, it was recently demonstrated how different neurological disorders may require different strategies aimed at MRI follow-up, highlighting the role of pre-operative planning [17]. Some critical considerations should be applied to our findings. Despite the highly positive correlation among the three observers, our findings were limited to observations made on two adult male volunteers: further data based on skull sizes of different dimensions (e.g., pediatric ones) should be contemplated. The unavailability of a 3 T MRI scanner at our institution limited our results to 1.5 T MRI-induced artifacts. A comparison among MRI artifacts produced by CI models with different diametrically bipolar magnets (e.g., Med-El Synchrony; Cochlear Nucleus Profile Plus) should be considered by further studies. When surgically implanted CI devices are investigated, MR imaging quality might show some differences compared to our external skin-surface position. Finally, additional artifacts produced by the intracochlear electrode array insertion should be further considered.

## Conclusions

The introduction of adaptive CI magnet systems has created a new scenario in the world of CI recipients. The need for post-surgical MRI surveillance may be overcome thanks to the adoption of strategies aimed at handling the MRI-induced artifacts. This study found that careful CI positioning allows for the visualization of various intracranial structures with sufficient visualization for diagnostic purposes, in case of unilateral CI. Bilateral CIs significantly deteriorate the anatomical visibility because of the two implant magnets' mutual interaction. What structures need to be viewed? Which MRI sequences are required? Answering these questions should now be part of pre-operative CI planning. CI positioning may play a crucial role in guiding the best surgical choice. However, further studies are mandatory to support and extend our research.

## Supplementary Information

Below is the link to the electronic supplementary material.

Supplementary file1 (TIF 3826 KB)

Supplementary file2 (TIF 1880 KB)

Supplementary file3 (TIF 1876 KB)

Supplementary file4 (TIF 1855 KB)

Supplementary file5 (TIF 1854 KB)

Supplementary file6 (TIF 1846 KB)

Supplementary file7 (TIF 1825 KB)

Supplementary file8 (TIF 1847 KB)

Supplementary file9 (TIF 1838 KB)
