# Supplementary figures and images for: The role of cochlear implant positioning on MR imaging quality: a preclinical in vivo study with a novel implant magnet system
Source: Eur Arch Otorhinolaryngol. 2021 Aug 9;279(6):2889–98. doi: 10.1007/s00405-021-07005-y (PMC9072450; doi:10.1007/s00405-021-07005-y)

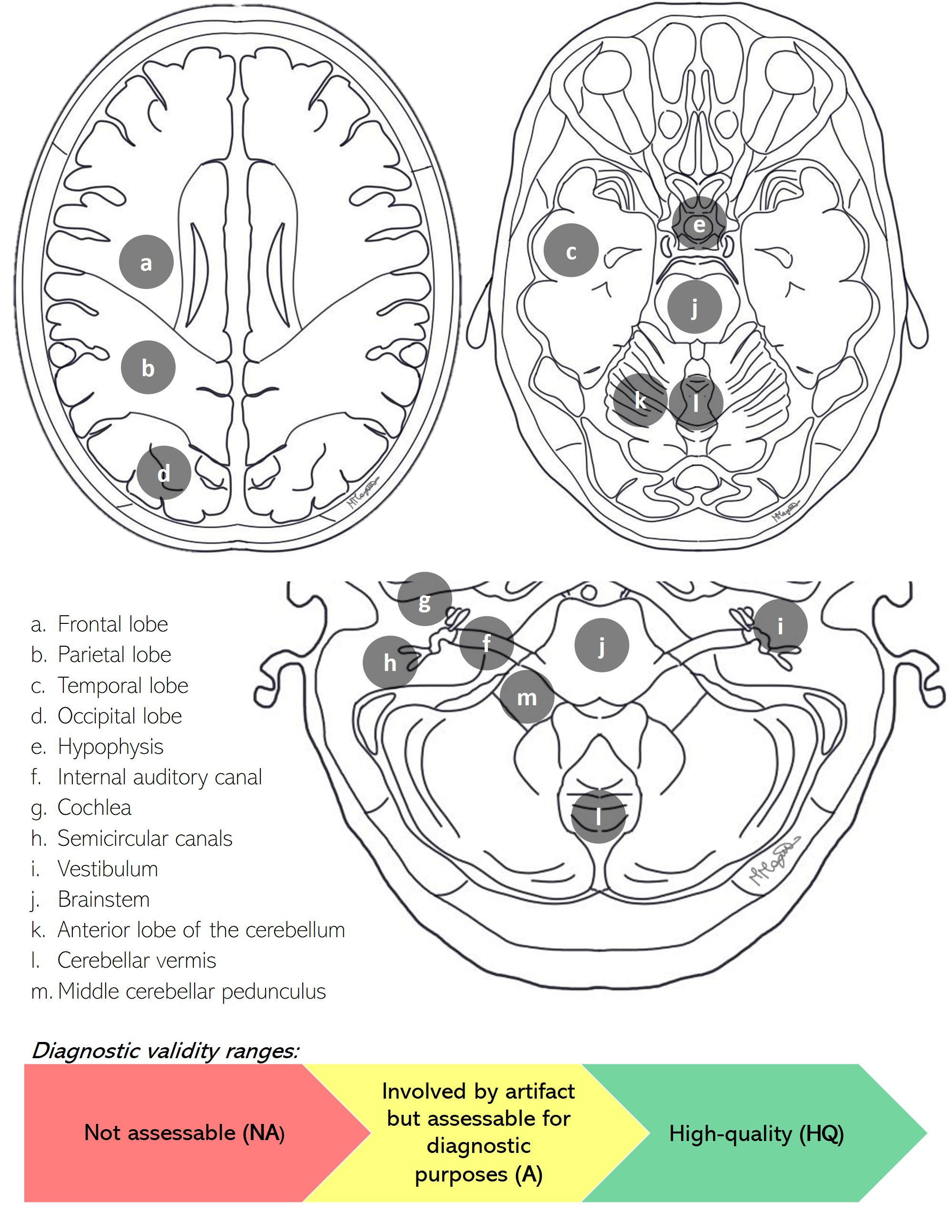

Supplement: Supplementary file 1 — Supplementary file1 (TIF 3826 KB) [file 405_2021_7005_MOESM1_ESM.tif]

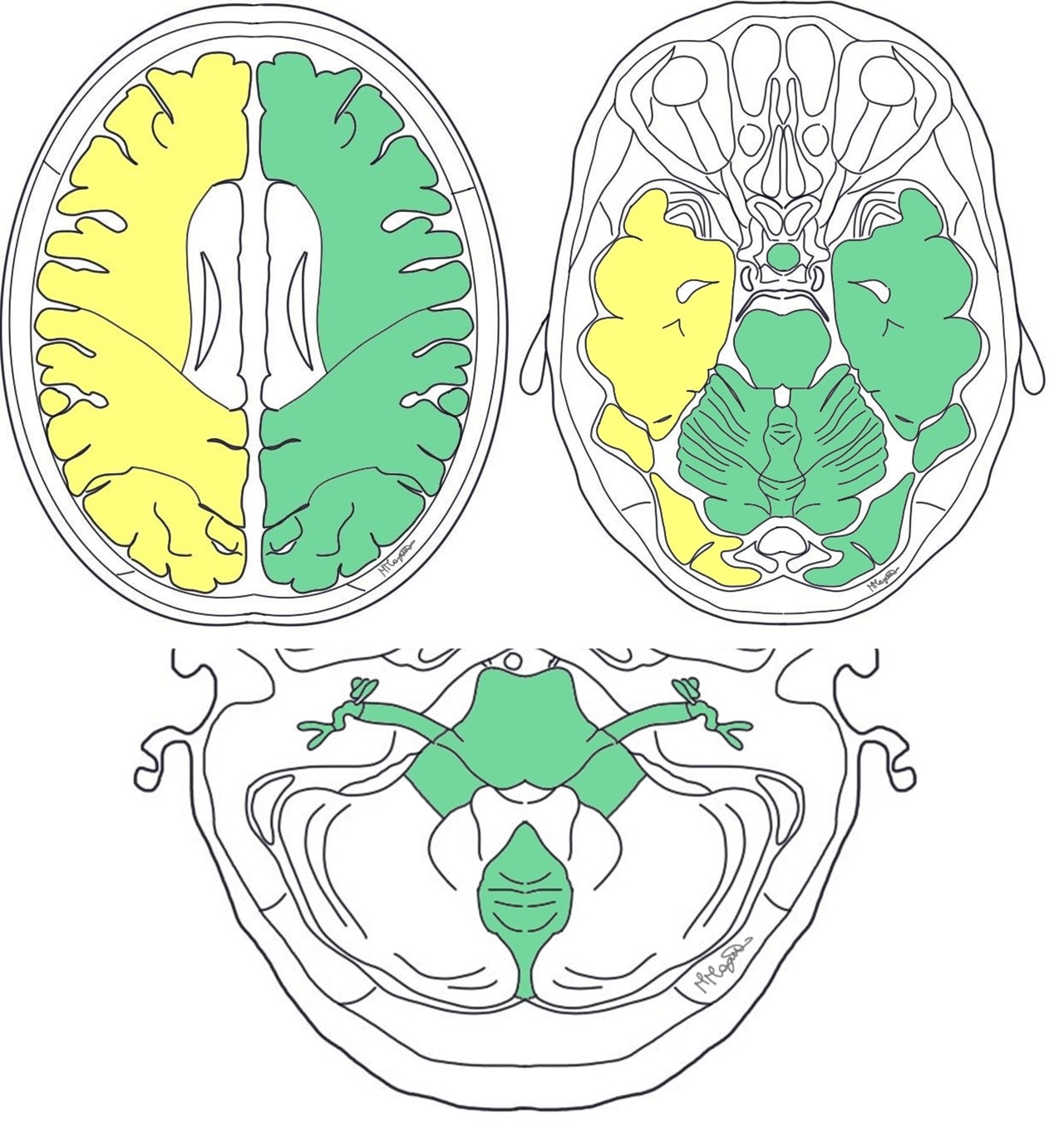

Supplement: Supplementary file 2 — Supplementary file2 (TIF 1880 KB) [file 405_2021_7005_MOESM2_ESM.tif]

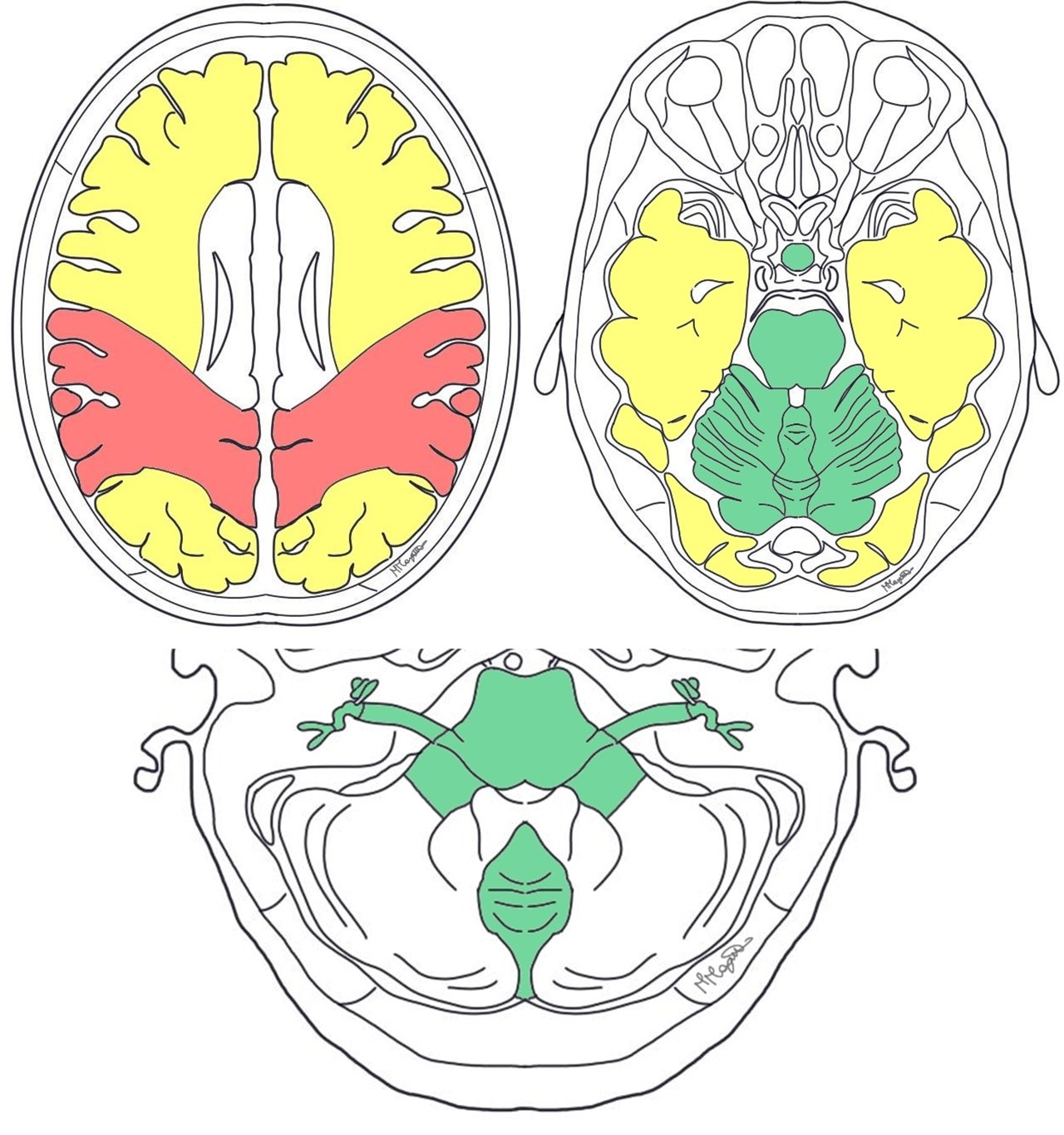

Supplement: Supplementary file 3 — Supplementary file3 (TIF 1876 KB) [file 405_2021_7005_MOESM3_ESM.tif]

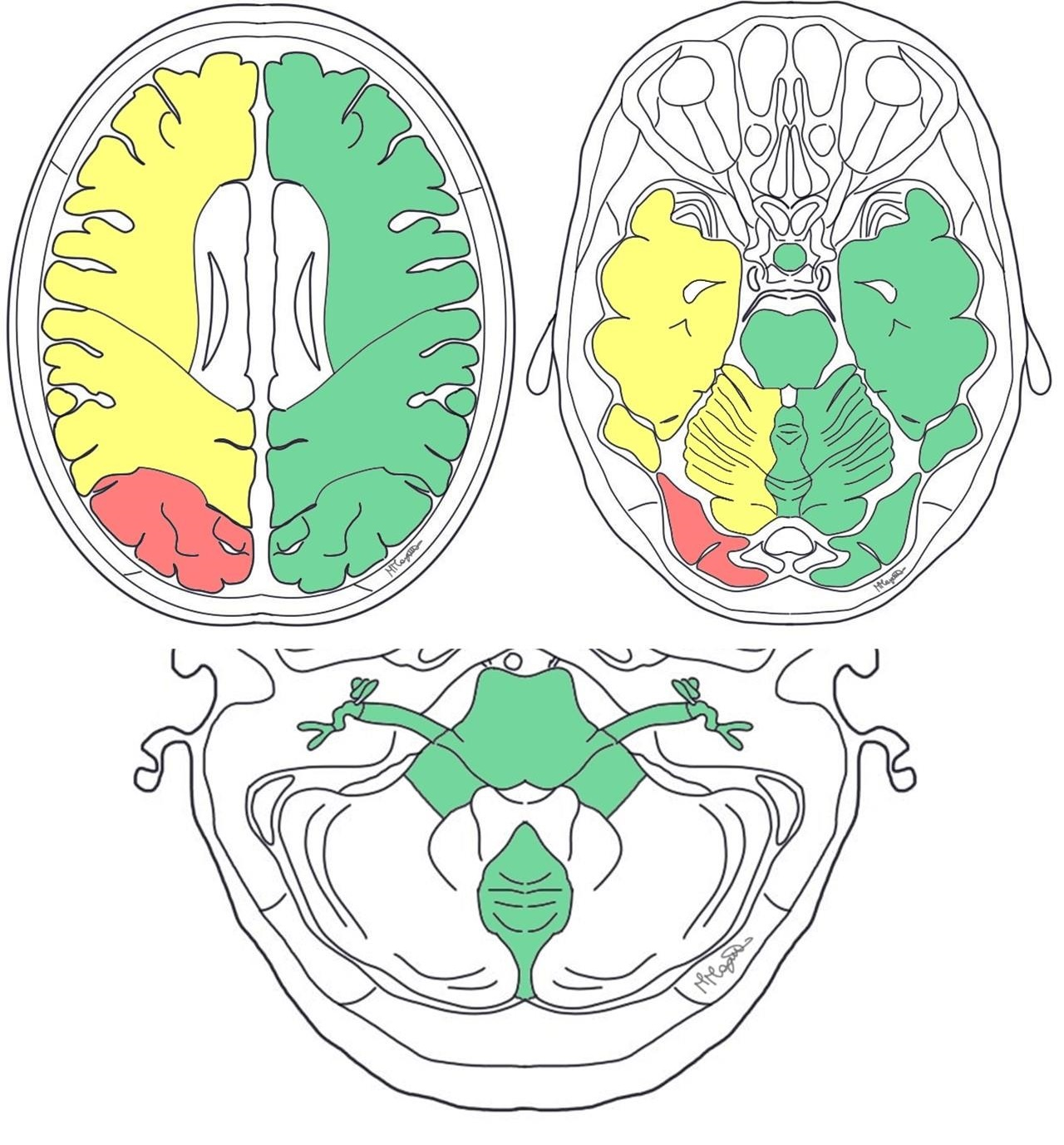

Supplement: Supplementary file 4 — Supplementary file4 (TIF 1855 KB) [file 405_2021_7005_MOESM4_ESM.tif]

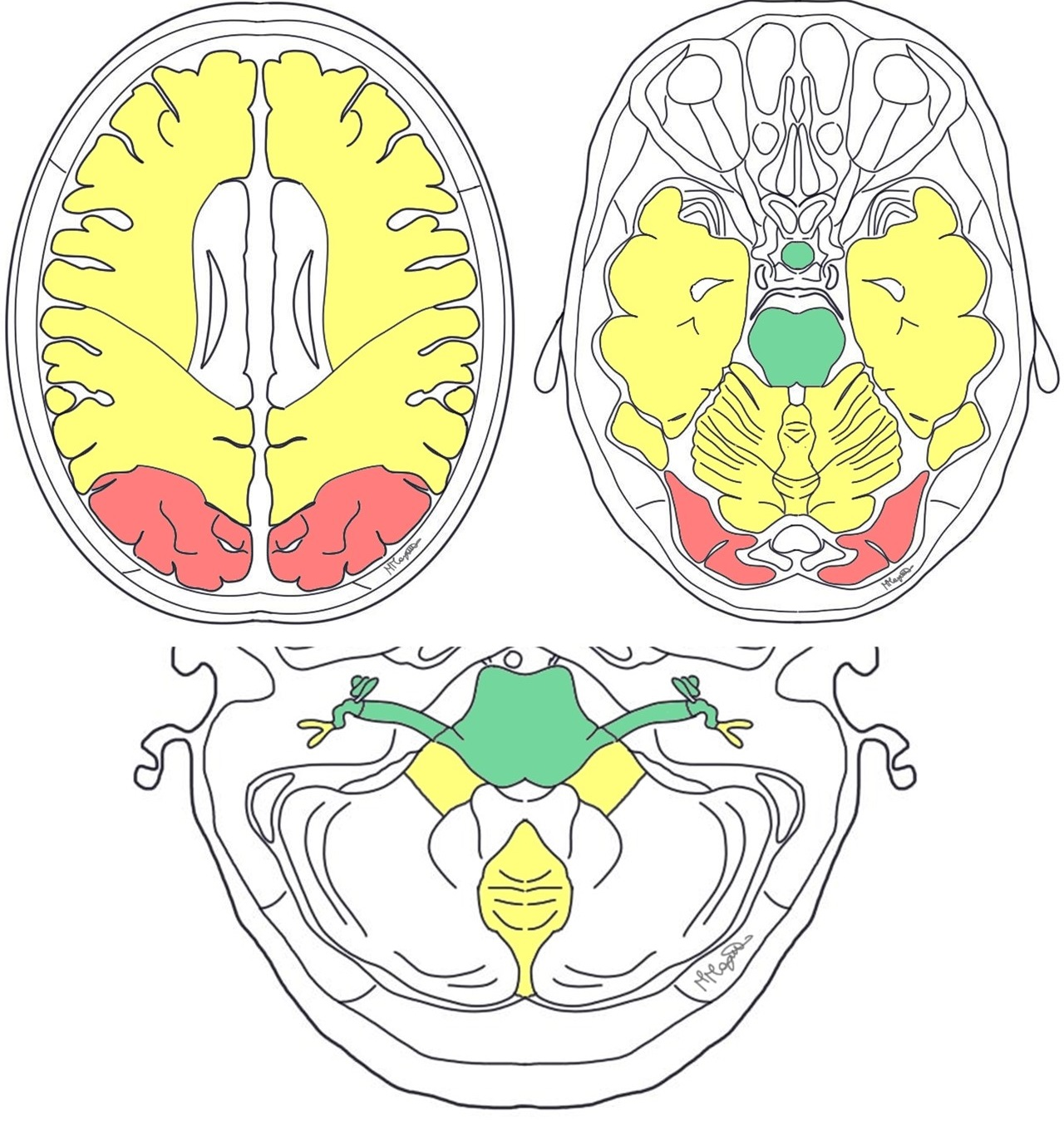

Supplement: Supplementary file 5 — Supplementary file5 (TIF 1854 KB) [file 405_2021_7005_MOESM5_ESM.tif]

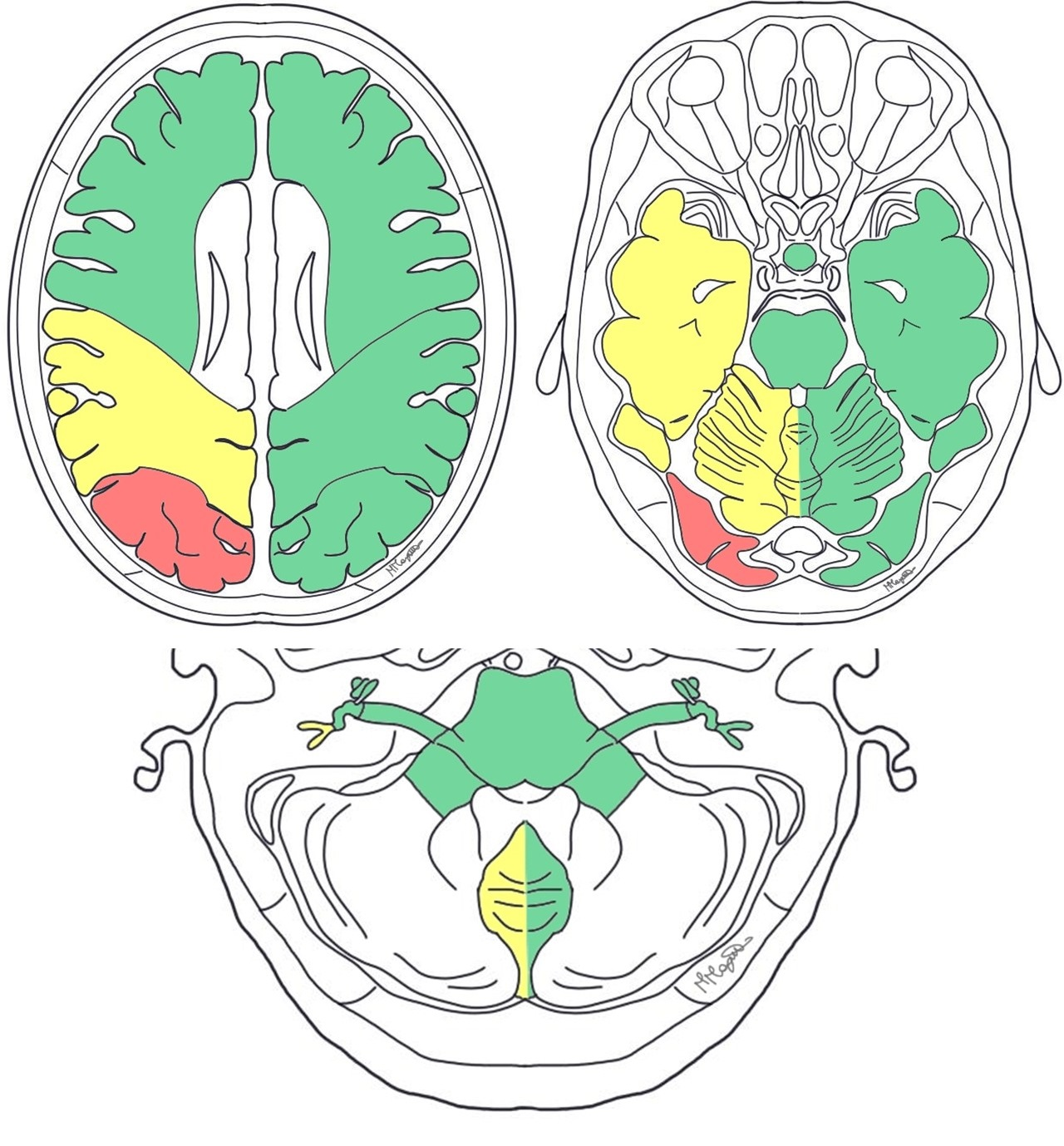

Supplement: Supplementary file 6 — Supplementary file6 (TIF 1846 KB) [file 405_2021_7005_MOESM6_ESM.tif]

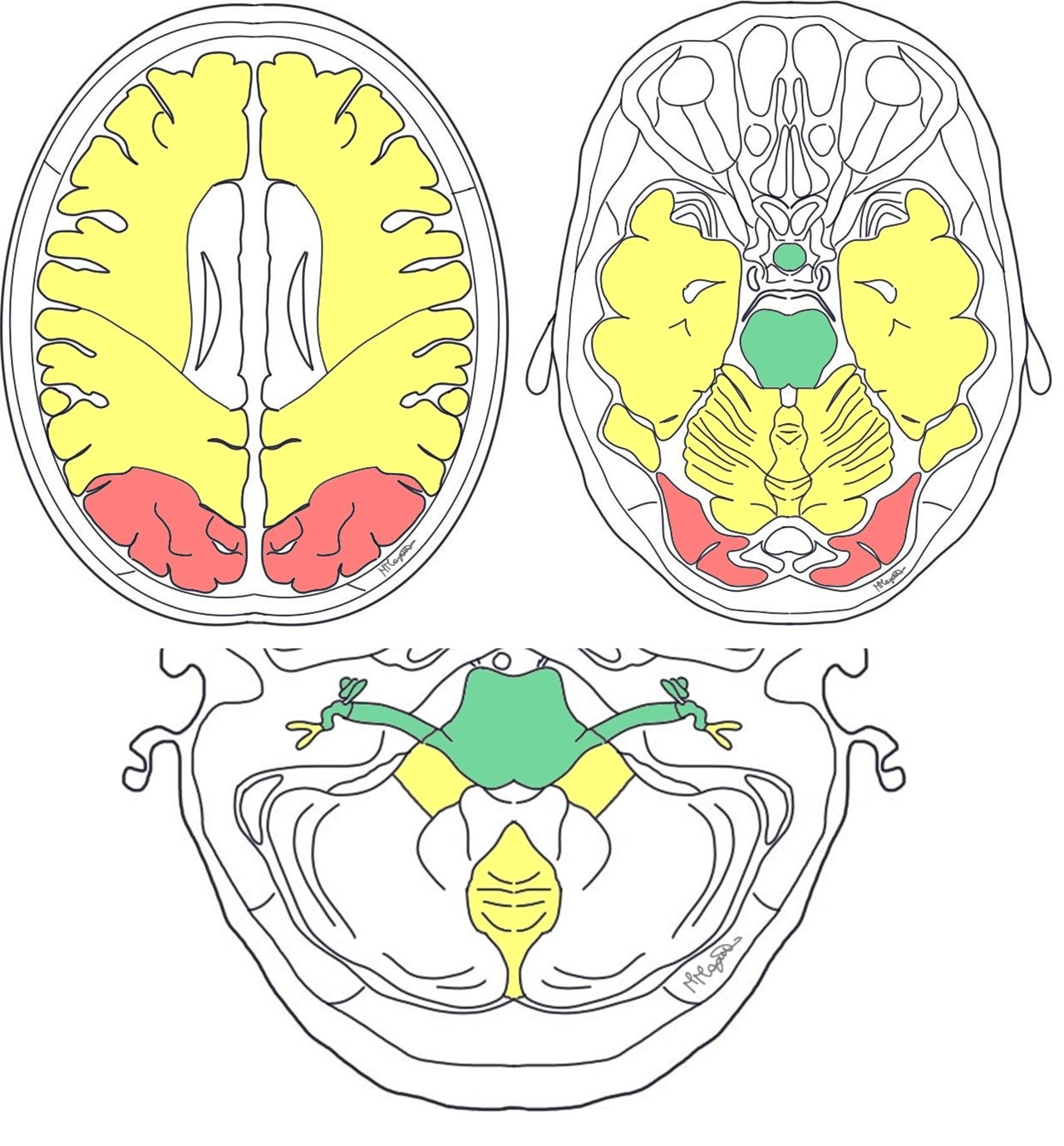

Supplement: Supplementary file 7 — Supplementary file7 (TIF 1825 KB) [file 405_2021_7005_MOESM7_ESM.tif]

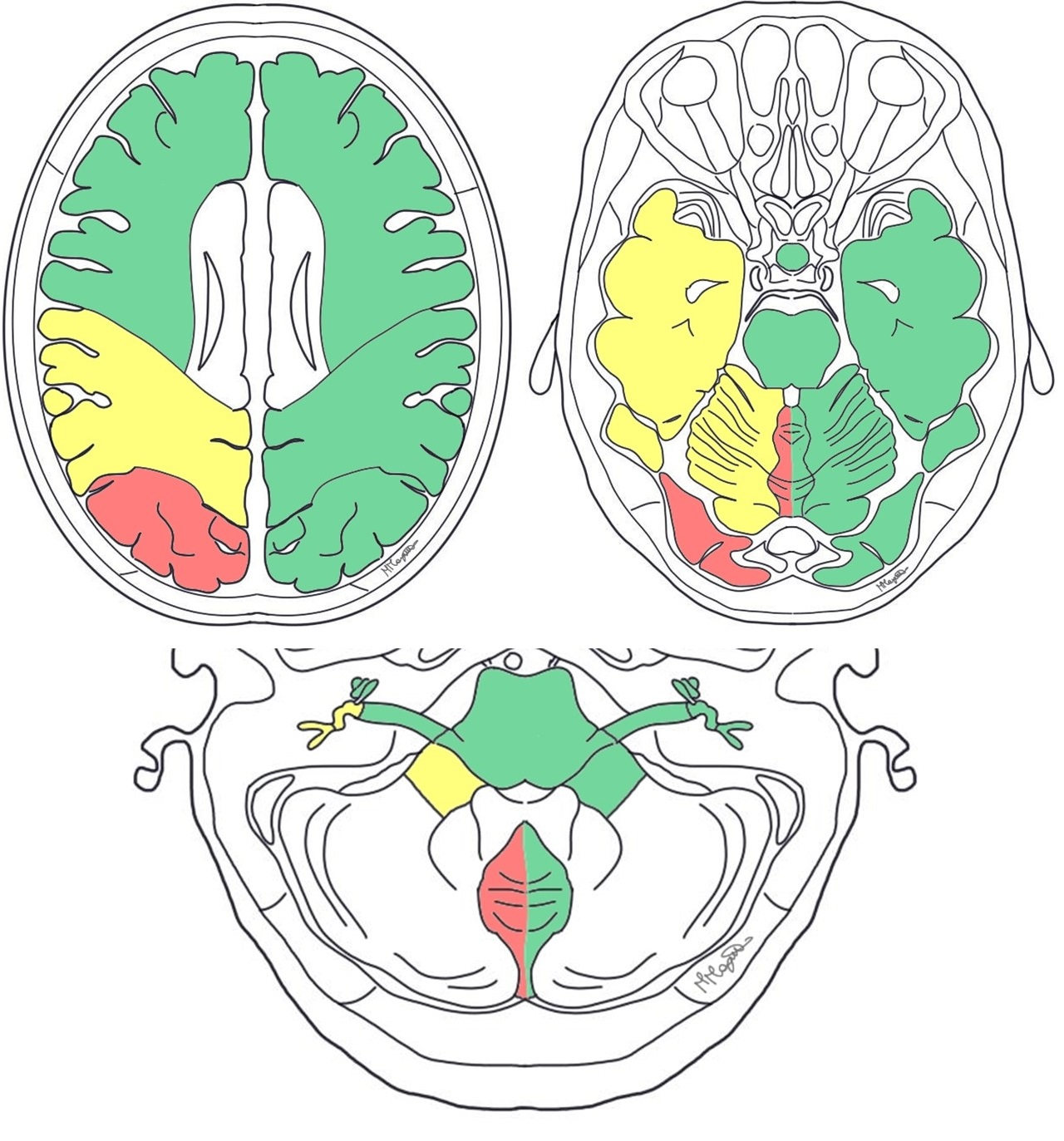

Supplement: Supplementary file 8 — Supplementary file8 (TIF 1847 KB) [file 405_2021_7005_MOESM8_ESM.tif]

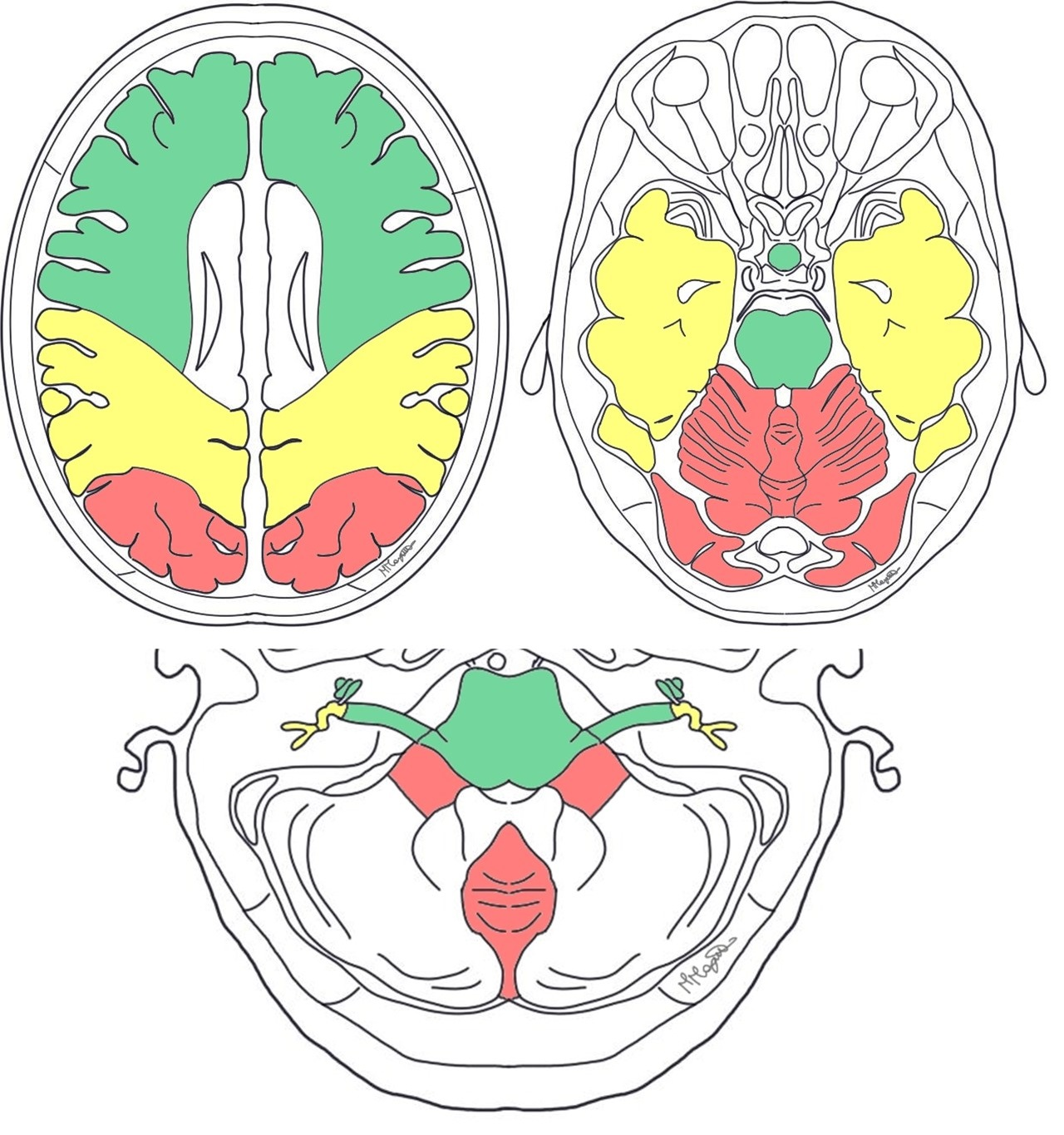

Supplement: Supplementary file 9 — Supplementary file9 (TIF 1838 KB) [file 405_2021_7005_MOESM9_ESM.tif]
